# *De novo* phasing with optimized XFEL data

**DOI:** 10.1107/S2052252516006758

**Published:** 2016-04-29

**Authors:** Quan Hao

**Affiliations:** a School of Biomedical Sciences, L04-48 Laboratory Block, University of Hong Kong, 21 Sassoon Road, Hong Kong

**Keywords:** serial crystallography, XFEL, SAD phasing

## Abstract

Nass *et al.* [*IUCrJ* (2016), **3**, 180–191] have demonstrated that serial femtosecond crystallography (SFX) data collected at X-ray free-electron lasers (XFELs) can be successfully phased using only the weak anomalous scattering from the native S atoms.

The unique capabilities of X-ray free-electron lasers (XFELs) have opened up new opportunities to collect damage-free diffraction data from randomly oriented sub-micron-sized crystals using serial femtosecond crystallography (SFX) (Chapman *et al.*, 2011 ▸). SFX data have been shown to be of sufficient quality to enable structure determination with the molecular replacement method (Kang *et al.*, 2015 ▸). However, *de novo* phasing with the SFX data is difficult because the fluctuations of intensity measurements may overwhelm the anomalous scattering signal owing to variation of crystal size, uneven photon spectral distribution, partial Bragg reflection and partial intersection of the crystal in the X-ray beam. In 2014, Schlichting and co-workers (Barends *et al.*, 2014 ▸) successfully utilized the strong anomalous signal of gadolinium to phase the single-wavelength anomalous diffraction (SAD) data collected at the Linac Coherent Light Source (LCLS) from crystals of a gadolinium derivative of lysozyme. In reality, however, finding a suitable derivative is often difficult. Therefore, use of endogenous atoms, such as sulfur, as a source of anomalous scattering would be a much more attractive approach. Nakane *et al.* (2015 ▸) demonstrated the native sulfur phasing of lysozyme from SFX data collected at SACLA.

In this issue of **IUCrJ**, Schlichting and co-workers (Nass *et al.*, 2016 ▸) report an optimized method to tackle the particular challenges of native sulfur phasing of SFX data collected at XFELs. They propose to (1) optimize the geometry (metrology) of the multi-tiled detector by translating and rotating individual tiles of the detector to minimize the distance between the predicted and observed spot locations, (2) refine the sample-to-detector distance by manually adapting the distance until the distributions of unit-cell lengths resemble single-Gaussian-like distributions, and (3) scale the integrated intensities from each diffraction image. They first tested their approach with the gadolinium-derivative lysozyme data and showed that the optimization improved the anomalous signal significantly: fully automated building of the structure with only 10 000 indexed images instead of the 60 000 images that were required in the original report (Barends *et al.*, 2014 ▸).

They then applied the same methodology to a thaumatin data set collected at the LCLS using the native sulfur as a source of anomalous scatterering (Fig. 1 ▸). A photon energy of 6 keV was chosen to balance the quest for a high anomalous signal of the relatively light atoms (sulfur) and thus low photon energy, and the limitations set by beamline transmission, detector quantum efficiency, the resolution of the diffraction data and photon absorption. They were able to phase SFX data using the weak anomalous signal of sulfur and automatically build an almost complete molecular model using 125 000 indexed patterns of thaumatin microcrystals.

X-ray free-electron lasers have provided a new way to obtain SFX data that is advantageous over traditional synchrotron sources in terms of better data resolution, smaller sample size and minimal radiation damage (Kang *et al.*, 2015 ▸). However, *de novo* phasing of SFX data, particularly with only native crystals, is still far from trivial. Millions of crystals and many hours of beamtime are usually required to collect a sufficient amount of data for structure determination. Nass *et al.*’s study has demonstrated that with optimized data processing technology, single-wavelength anomalous diffraction data from the native sulfur atoms can allow protein structures to be solved *ab initio*. Improvements in XFEL instrumentation and processing software should further reduce the number of images required to phase SFX data.

## Figures and Tables

**Figure 1:**
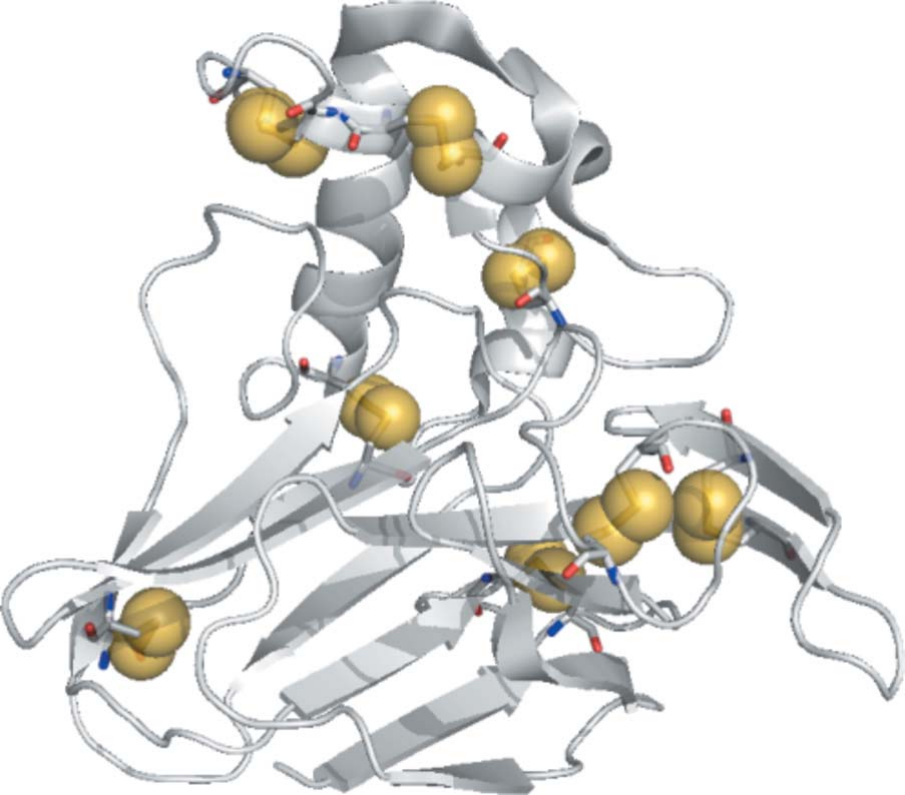
Single-wavelength anomalous diffraction from the sulfur atoms (colored in yellow) in native thaumatin was used to phase SFX data.
